# New Conjugates of Vancomycin with Cell-Penetrating Peptides—Synthesis, Antimicrobial Activity, Cytotoxicity, and BBB Permeability Studies

**DOI:** 10.3390/molecules29235519

**Published:** 2024-11-22

**Authors:** Jarosław Ruczyński, Katarzyna Prochera, Natalia Kaźmierczak, Katarzyna Kosznik-Kwaśnicka, Lidia Piechowicz, Piotr Mucha, Piotr Rekowski

**Affiliations:** 1 Laboratory of Chemistry of Biologically Active Compounds, Faculty of Chemistry, University of Gdańsk, Wita Stwosza 63, 80-308 Gdańsk, Poland; katarzyna.prochera@phdstud.ug.edu.pl (K.P.); piotr.mucha@ug.edu.pl (P.M.); piotr.rekowski@ug.edu.pl (P.R.); 2 Department of Medical Microbiology, Faculty of Medicine, Medical University of Gdańsk, Dębowa 25, 80-204 Gdańsk, Poland; natalia.lubowska@gumed.edu.pl (N.K.); katarzyna.kwasnicka@gumed.edu.pl (K.K.-K.); lidia.piechowicz@gumed.edu.pl (L.P.)

**Keywords:** vancomycin, antimicrobial peptides, cell-penetrating peptides, Van-CPP conjugates, antimicrobial assay, cytotoxicity assay, PAMPA-BBB assay, click chemistry

## Abstract

Vancomycin (Van) is a glycopeptide antibiotic commonly used as a last resort for treating life-threatening infections caused by multidrug-resistant bacterial strains, such as *Staphylococcus aureus* and *Enterococcus* spp. However, its effectiveness is currently limited due to the rapidly increasing number of drug-resistant clinical strains and its inherent cytotoxicity and poor penetration into cells and specific regions of the body, such as the brain. One of the most promising strategies to enhance its efficacy appears to be the covalent attachment of cell-penetrating peptides (CPPs) to the Van structure. In this study, a series of vancomycin conjugates with CPPs—such as TP10, Tat (47–57), PTD4, and Arg_9_—were designed and synthesized. These conjugates were tested for antimicrobial activity against four reference strains (*Staphylococcus aureus*, *Enterococcus faecalis*, *Escherichia coli*, and *Pseudomonas aeruginosa*) and two clinical drug-resistant strains: methicillin-resistant *S. aureus* and vancomycin-resistant *E. faecium*. In addition, cytotoxicity tests (using a human fibroblast cell line) and blood–brain barrier (BBB) permeability tests (using a parallel artificial membrane permeability assay—PAMPA-BBB assay) were conducted for selected compounds. Our research demonstrated that conjugation of Van with CPPs, particularly with Tat (47–57), Arg_9_, or TP10, significantly enhances its antimicrobial activity against Gram-positive bacteria such as *S. aureus* and *Enterococcus* spp., reduces its cytotoxicity, and improves its access to brain tissues. We conclude that these findings provide a strong foundation for the design of novel antimicrobial agents effective in treating infections caused by drug-resistant staphylococcal and enterococcal strains, while also being capable of crossing the BBB.

## 1. Introduction

Bacterial infections are among the leading causes of death worldwide [1,2,3]. The extensive and often inappropriate use of antibiotics has led to a rapid rise in bacteria resistant to known antimicrobial agents [2,3]. In recent years, antibiotic resistance has become a major public health concern [3]. Current commercially available antibiotics are insufficient to meet the growing demand for therapies targeting multidrug-resistant (MDR) bacteria. Consequently, there is an urgent need to develop new compounds capable of effectively combating the continually evolving MDR bacteria. Various approaches have been proposed to address bacterial infections, including monoclonal antibodies, antimicrobial peptides (AMPs), quorum-sensing inhibitors, bacteriophages, and metal-based or polymer-based nanoparticles [4]. Other strategies focus on modifying the structure of known antibiotics, such as vancomycin, to enhance their efficacy or alter their in vivo properties.

Vancomycin (Van) is a hydrophilic glycopeptide antibiotic with bactericidal activity against Gram-positive aerobes and anaerobes [5]. Van is often used as a last-resort treatment for life-threatening infections caused by MDR bacteria such as *Staphylococcus aureus*, *Enterococcus* spp., and *Clostridium difficile* [1,6,7]. Currently, these microorganisms are the leading causes of community-acquired infections that result in high morbidity and mortality rates. Van inhibits cell wall synthesis by binding to the *C*-terminal d-Ala-d-Ala of pentapeptide precursors in peptidoglycan (lipid II), thus blocking the transglycosylation and transpeptidation reactions essential for cell wall biosynthesis [2]. Despite its therapeutic significance, Van has limitations. One major concern is the emergence of resistance among *S. aureus* strains resistant to methicillin (MRSA) and vancomycin (VISA or VRSA), as well as vancomycin-resistant enterococcal strains (VRE) [7,8]. In recent years, several types of Van resistance have been described, of which *VanA* type was the first to be elucidated and is the most common [9]. Replacement of the d-Ala-d-Ala sequence with d-Ala-d-Lac (*VanA* and *VanB* type of resistance) or d-Ala-d-Ser (*VanC*, *VanE*, and *VanG* type of resistance) causes Van not to bind to the peptidoglycan precursors so that peptidoglycan can be synthesized and the cell wall can be formed [1,2,5,6,7,8,10,11]. Moreover, vancomycin exhibits toxic effects (ototoxicity, nephrotoxicity, and hypersensitivity reactions) [11,12] and, as a consequence of its hydrophilic nature, it has poor access to the cell interior or specific regions of the body, such as the brain [7,11].

To address these shortcomings, various strategies have been developed, including chemical modification of the vancomycin framework, synthesis of new derivatives, or conjugation with other chemical compounds [4,6,10,11,13,14,15,16,17]. Modifications to the core structure of vancomycin are particularly challenging due to its complexity and often require the total synthesis of vancomycin analogs [1,6]. More details on the total synthesis of vancomycin-related glycopeptide antibiotics, including their modifications and key analogs, can be found elsewhere [16,18].

Vancomycin is most commonly modified through a semisynthetic approach, targeting the *N*-terminus or *C*-terminus of the heptapeptide backbone or the vancosamine group within the carbohydrate moiety [4,6]. These modification sites are considered nonbinding regions and typically do not affect vancomycin’s binding affinity with the terminal residues of lipid II. Promising modification strategies include the introduction of cationic sulfonium moieties, single cationic quaternary ammonium charges combined with saturated fatty acids, as well as chlorobiphenyl and dipicolyl extensions [4,6,10,13,15]. Hydrophobic (lipophilic) substitutions at the vancosamine group, such as lipidation, enhance binding affinity to the lipid II termini and improve membrane anchoring [6]. On the other hand, the introduction of hydrophilic groups like sugar moieties results in enhanced efficacy, improved pharmacokinetics, and reduced toxicity [1]. Further approaches have focused on the conjugation of vancomycin with other biomolecules, such as fatty acids, single amino acids (e.g., arginine), carbohydrates, antimicrobial peptides (e.g., nisin, CRAMP, Hecate, polymyxin, polylysine, polyarginine), and lipopeptides [1,2,3,4,10,11,13,14,15,16,17,19,20,21].

In recent years, significant attention has been given to cell-penetrating peptides (CPPs). These short peptides, composed of up to 30 amino acids, serve as highly effective cellular delivery vectors due to their ability to cross cell membranes and the blood–brain barrier (BBB) [15,17,22,23,24,25]. CPPs not only gain access to the cell interior but also facilitate the delivery of various cargos (including fluorophores, plasmids, DNA, siRNA, PNA, proteins, peptides, and low-molecular-weight drugs) to their intended targets, such as mammalian cells or brain tissues, without damaging cell membranes. The precise mechanisms by which CPPs are translocated across biological membranes remain unclear. However, it is known that they can involve versatile endocytotic (endocytosis and pinocytosis) or non-endocytotic pathways (such as the “inverted micelle”, “barrel-stave”, “toroidal”, or “carpet” models) [17,22,23]. The mechanism applied depends on the chemical properties and molecular size of the cargo component, as well as the type of cell it will enter.

Moreover, some CPPs (e.g., Tat, penetratin, TP10, oligoarginine, or Pep-1) exhibit antimicrobial activity, largely due to their overall positive charge (derived from multiple positively charged amino acid residues such as arginine or lysine) and, in some cases, amphipathic properties (the presence of hydrophobic amino acid residues) [7,15,17].

These unique properties of certain CPPs (both penetrating and antimicrobial) suggest that the covalent attachment of CPPs to existing antibiotics represents one of the most promising strategies to enhance antimicrobial activity. Among known antibiotics, vancomycin appears to be an ideal candidate for conjugation with CPPs. Van-CPP conjugates could prove effective against multidrug-resistant bacterial strains with minimal mammalian cytotoxicity [7,15,17]. In addition, these dual-function compounds may be effective against intracellular pathogens and central nervous system (CNS) infections, exhibit high antibiofilm activity, stability in human serum, and potentially reduced immunogenicity.

Our recent studies have demonstrated that conjugates of vancomycin with transportan 10 (TP10) show enhanced antibacterial activity compared to unmodified vancomycin, particularly against clinical strains of MRSA and h-VISA, and in some cases, against VRE [7]. Notably, these conjugates exhibited antibacterial activity against intracellular MRSA in HEK293 cells and improved access to brain tissue without a significant increase in toxicity (as measured by erythrocyte lysis assay).

**Figure 1:**
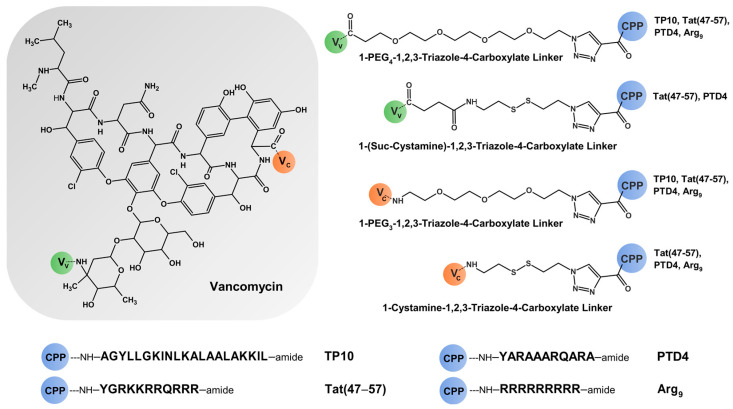
Schematic illustration of the chemical structure of the synthesized Van-CPP conjugates, where *V*_V_ is the primary amino position in the carbohydrate moiety of vancomycin (green circle, position of PEG_4_ or Suc-Cystamine linker attachment), *V*_C_ is the *C*-terminal carboxylic position of vancomycin (orange circle, position of PEG_3_ or Cystamine linker attachment), PEG_4_ is 4,7,10,13-tetraoxopentadecane-1-caboxylate, PEG_3_ is 1-amino-3,6,9-trioxoundecane, Cystamine is 1-amino-2-(ethyldisulfanyl)ethan, Suc-Cystamine is 4-(2-(ethyldisulfanyl)ethylamino)-4-oxobutane-1-carboxylate, CPP is the *N*-terminal amino position of cell-penetrating peptides (blue circle, position of CPP attachment): Tat (47–57) and PTD4 (attached via PEG_3_, PEG_4_, Cystamine and Suc-Cystamine linker), Arg_9_ (attached via PEG_3_, PEG_4_ and Cystamine linker) or TP10 (attached via PEG_3_ and PEG_4_ linker).

In the present work, we designed and synthesized a series of vancomycin conjugates with cell-penetrating peptides (CPPs), including TP10, Tat (47–57), PTD4, and Arg_9_ (Figure 1). TP10 is a 21-residue chimeric amphipathic CPP (a truncated analog of transportan), composed of the *N*-terminal fragment (7–12) of the neuropeptide galanin, linked via a Lys residue to the full-length wasp venom peptide mastoparan [7,22,23,24,25,26]. It contains four basic lysine residues, giving it a total charge of +5 at physiological pH. TP10 is recognized not only for its ability to transport various cargos across cell membranes but also for its antimicrobial activity against Gram-positive bacteria, including *S. aureus*, although cytotoxicity may occur at higher concentrations.

Another CPP used in this study was Tat (47–57), an arginine-rich motif derived from the transcription factor of the HIV-1 Tat protein [15,22,23]. This 11-residue cationic peptide, with a total charge of +9, has been shown to internalize in human cells and also demonstrates broad-spectrum antimicrobial activity against *E. coli*, *S. aureus*, and *B. subtilis*. The observation that the Tat peptide penetrates mammalian cells due to its highly basic nine-amino acid domain inspired the design of a new group of polycationic CPPs, including oligoarginines such as Arg_9_. Arg_9_ is a short nine-residue peptide with a total charge of +10, known for its cell-penetrating properties and antimicrobial activity against Gram-positive bacterial cells (including *S. aureus*), with relatively low cytotoxicity against eukaryotic cells [22,23,27].

In contrast, PTD4 is a less basic alanine-scan analog of the Tat (47–57) fragment, with a total charge of +4 and 33-fold higher penetrating ability than the original peptide; however, its antibacterial activity is unknown [28]. The above-mentioned CPPs were covalently coupled to vancomycin with the use of the highly efficacious and chemoselective 1,3-dipolar Huisgen’s cycloaddition (known as the “click reaction”) and various linkers containing polyethylene glycol (PEG_3_ or PEG_4_) or a disulfide bridge (Cystamine or Suc-Cystamine). The obtained conjugates as well as their parent components (TP10, Tat (47–57), PTD4, and Arg_9_) were investigated concerning their antimicrobial activity (against several bacterial strains, including MRSA and VRE), cytotoxicity (using human fibroblast cell line) and BBB permeability (using parallel artificial membrane permeability assay—PAMPA-BBB assay). As a result of our studies, we expected to find new compounds with enhanced antibacterial activity, low cytotoxicity, and the ability to cross biological membranes such as the blood–brain barrier.

## 2. Results

### 2.1. Antimicrobial Activity

A comparison of the MIC values for the tested compounds, which reflect their antimicrobial activity against three reference bacterial strains (*S. aureus*, *E. faecalis*, and *E. coli*) as well as clinical strains of MRSA and VRE, is presented in Table 1. Given the significant differences in molecular mass between the compounds, all MIC values are expressed in both μM and μg/mL. However, to more accurately represent their action, molar concentrations (μM) were selected for the presentation of the MIC values. Vancomycin (a glycopeptide antibiotic) and gentamicin (an aminoglycoside antibiotic) were used as controls in this study. The MIC values for *P. aeruginosa* are not included in Table 1, as all tested compounds, except gentamicin (with a MIC of 4.08 µM), showed no inhibitory activity against this strain at the concentrations tested.

As shown in Table 1, all examined strains were susceptible to gentamicin (with MIC values ranging from 0.5 µM to 16.3 µM). When exposed to vancomycin, only *S. aureus* (including MRSA) and *E. faecalis* strains were susceptible, with MICs of 1.35, 1.01, and 5.38 µM, respectively. The clinical strain of *E. faecium* as well as the reference strains of *E. coli* and *P. aeruginosa* were resistant to Van. The attachment of TP10 via a PEG_3_ or PEG_4_ linker to the vancomycin molecule changed its antibacterial activity. The Van-PEG_3_-TP10 conjugate (**2**) showed slightly enhanced activity compared to unmodified Van against the reference strains *S. aureus* (with MIC = 1.03 µM) and *E. faecalis* (with MIC = 4.12 µM), and relatively high activity against *E. coli* (with MIC = 1.03 µM), for which Van is inactive. The clinical MRSA strain was not susceptible to treatment with this conjugate. However, Van-PEG_4_-TP10 (**3**) was more active than Van only against *E. faecalis* (with MIC = 4.04 µM) and *E. coli* (with MIC = 8.09 µM) strains. TP10 (**1**), the CPP component of the conjugates, turned out to be less active than Van against the tested bacterial strains (except *E. coli*), showing MIC values ranging from 3.67 to 29 µM.

Attachment of the Tat (47–57) peptide to the vancomycin core using different linkers (such as PEG_3_, PEG_4_, Cystamine, or Suc-Cystamine) proved advantageous by improving Van’s antibacterial activity. All Van-Tat conjugates (**5**–**8**) exhibited higher antibacterial activity than Van alone, particularly against the reference strains *S. aureus* (with MIC values ranging from 0.077 to 0.3 µM) and *E. faecalis* (with MIC values ranging from 0.15 to 1.20 µM). However, the clinical strain of MRSA was less susceptible to these conjugates than to vancomycin. Among the conjugates, only Van-Suc-Cystamine-Tat (47–57) (**8**) showed higher inhibitory activity against MRSA (with an MIC of 0.60 µM). The Van-resistant clinical strain of *E. faecium* also exhibited susceptibility to Van-Tat conjugates, with Van-PEG_3_-Tat (47–57) (**5**) having the lowest MIC value of 0.31 µM. Prop-Tat (47–57) (**4**), the CPP component of the conjugates, was less active than Van against the tested bacterial strains, with only a relatively low MIC value of 4.97 µM against the reference strain *S. aureus*. However, *E. coli* and *P. aeruginosa* strains were resistant to these compounds.

Among the conjugates of vancomycin with PTD4 (a less basic Ala-scan analog of the Tat (47–57) fragment), two—Van-PEG_3_-PTD4 (**10**) and Van-PEG_4_-PTD4 (**11**)—demonstrated inhibitory activity comparable to Van against the reference strains *S. aureus* (with MICs of 1.38 and 1.34 µM, respectively) and *E. faecalis* (with MICs of 5.51 and 5.37 µM, respectively). However, when tested against MRSA, these compounds were less potent inhibitors of bacterial growth compared to unmodified vancomycin (with MICs of 2.76 and 2.69 µM, respectively). Van-Cystamine-PTD4 (**12**), another conjugate in this group, showed lower MIC values than vancomycin against both the MRSA strain (with an MIC of 0.70 µM) and the reference strain *E. faecalis* (with an MIC of 2.79 µM). However, the vancomycin-resistant *E. faecium* strain, as well as *E. coli* and *P. aeruginosa*, were resistant to these conjugates. Unlike other CPP components, Prop-PTD4 (**9**) was completely inactive against all tested strains.

The conjugation of the polycationic Arg_9_ peptide with vancomycin using PEG_3_, PEG_4_, or Cystamine linkers resulted in conjugates with high antibacterial activity against staphylococcal and enterococcal strains. One conjugate, Van-PEG_3_-Arg_9_ (**15**), exhibited strong inhibitory activity against the MRSA strain (MIC = 0.16 µM), the reference strain of *E. faecalis* (MIC = 0.64 µM), and the VRE strain of *E. faecium* (MIC = 0.64 µM). Interestingly, the reference strain of *S. aureus* was not susceptible to this compound. Another conjugate, Van-PEG_4_-Arg_9_ (**16**), was particularly effective against both the reference strain of *S. aureus* and the MRSA strain (both with MICs of 0.078 µM), as well as against the reference strain of *E. faecalis*. However, the Van-Cystamine-Arg_9_ conjugate (**17**) demonstrated only moderate inhibitory activity against the reference strains of *S. aureus* and *E. faecalis* (with MICs of 1.30 and 2.59 µM, respectively). Strains of *E. coli* and *P. aeruginosa* were resistant to these conjugates. The CPP component, Prop-Arg_9_ (**14**), was found to be less active than Van against the tested strains (except for the VRE strain), with MIC values ranging from 2.71 to 87 µM.

#### Interactions Between Van and CPP Components of the Conjugates

To assess possible interactions between Van and the CPP components of the conjugates (such as Prop-Tat (47–57) and Prop-Arg_9_), fractional inhibitory concentration indexes (FICI) were determined. As shown in Table 2, the calculated FICI values for the tested *S. aureus* strain (ATCC 29213) were 3.75 and 2.53 for the combinations of Van with Prop-Tat (47–57) and Van with Prop-Arg_9_, respectively. These values indicate that neither synergy nor antagonism occurs between Van and Prop-Tat (47–57) or Van and Prop-Arg_9_. Values of FICI > 0.5 and ≤4 reflect indifference, meaning no significant interaction. However, the interaction between Van and TP10 (as investigated in previous studies [7]) or between Van and Prop-PTD4 (due to the lack of antibacterial activity of Prop-PTD4) was not tested in the current study.

### 2.2. Cytotoxicity

**Figure 2:**
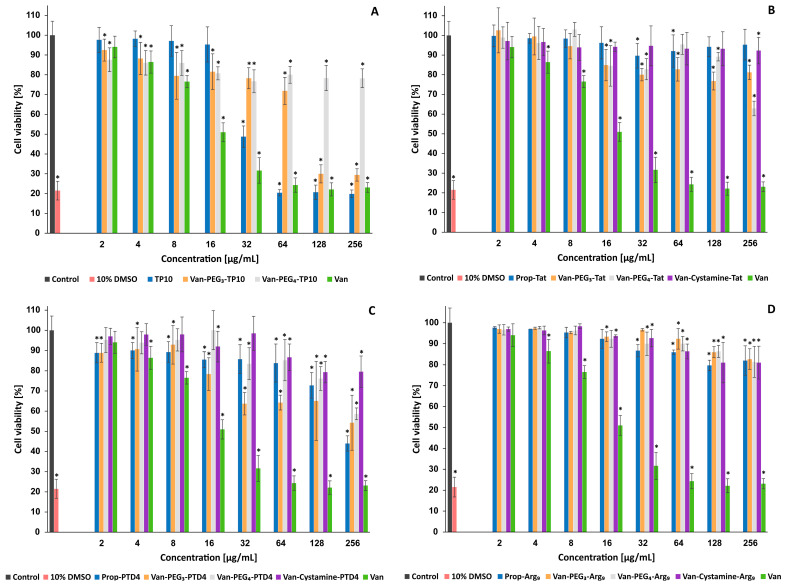
Effects of vancomycin conjugates and their components on the viability of normal human fibroblast cells: (**A**) TP10 and its conjugates, (**B**) Tat(47–57) and its conjugates, (**C**) PTD4 and its conjugates, (**D**) Arg_9_ and its conjugates. Cells were incubated with various concentrations of peptides for 24 h and cell viability was assessed by neutral red cytotoxicity assay. Plots present mean ± SD from three independent experiments performed in triplicate. The x-axis represents peptide concentration in µg/mL. The y-axis represents cell viability expressed as a percentage relative to the untreated control cells incubated without peptides as well as control cells treated with 10% DMSO. A one-way ANOVA test was used to test the degree of significance. * statistically significant (*p* < 0.05) as compared to control.

The cytotoxicity of the Van-CPP conjugates, as well as their components, was evaluated against normal human fibroblast cells. Cell viability was assessed using the neutral red cytotoxicity assay after incubation with various concentrations of the tested compounds (Figure 2). The fibroblast cell line showed sensitivity to 10% DMSO (used as a positive control), with cell viability reduced to approximately 21% after 24 h of incubation.

Our studies demonstrated that vancomycin, at concentrations up to 4 µg/mL, did not substantially affect cell viability. However, at concentrations above 16 µg/mL, cell viability significantly decreased to approximately 32–22% in the concentration range of 32 to 256 µg/mL. In contrast, both Van conjugates with TP10 (**2**, **3**) exhibited significantly lower cytotoxicity across the tested concentration ranges (Figure 2A). Van-PEG_3_-TP10 (**2**) showed notable cytotoxicity only at concentrations above 64 µg/mL (with cell viability around 30%), while Van-PEG_4_-TP10 (**3**) maintained about 78% cell viability even at 256 µg/mL. TP10 (**1**), the CPP component of these conjugates, demonstrated lower cytotoxicity than vancomycin but higher than the Van-TP10 conjugates, with cell viability dropping to around 20% at concentrations above 32 µg/mL.

Similarly, the three Van-Tat (47–57) conjugates (**5**–**7**) and their CPP component (Prop-Tat (47–57) peptide, **4**) did not significantly affect cell viability across the tested concentration range, except for Van-PEG_4_-Tat (47–57) (**6**), which reduced cell viability to about 63% at 256 µg/mL (Figure 2B). For the vancomycin conjugates with PTD4 (a less basic analog of the Tat (47–57) peptide) and their CPP component (analog Prop-PTD4), the cytotoxic effect was less pronounced (Figure 2C). Of the three conjugates tested, only Van-Cystamine-PTD4 (**12**) did not significantly affect cell viability. The other two conjugates, Van-PEG_3_-PTD4 (**10**) and Van-PEG_4_-PTD4 (**11**), showed some cytotoxicity at concentrations above 16 and 64 µg/mL, respectively, with cell viability ranging between 55 and 58% at 256 µg/mL. In contrast, the alkyne-functionalized Prop-PTD4 peptide (**10**) demonstrated significantly greater cytotoxicity than its vancomycin conjugates, reducing cell viability to about 44% at 256 µg/mL.

Finally, the Van conjugates with oligoarginine (Arg_9_) (**15**–**17**) and their CPP component (Prop-Arg_9_, **14**) showed minimal cytotoxicity across the tested concentrations (Figure 2D). For these compounds, cell viability decreased slightly to around 82% at 256 µg/mL, indicating relatively low cytotoxicity.

### 2.3. BBB Permeability

To predict the ability of the synthesized conjugates to diffuse through the blood–brain barrier (BBB), the PAMPA-BBB assay was used as a noncell-based method for measuring the passive permeability of selected compounds. In this study, artificial membranes mimicking the BBB were prepared using porcine polar brain lipid extract. As expected, diazepam (used as the high-permeability control) demonstrated good brain permeability, with a permeation coefficient (P*_e_*) value significantly higher than 4.0 × 10^−6^ cm/s, while diclofenac (used as the low-permeability control) showed rather uncertain BBB permeation (P*_e_* value of 3.44 × 10^−6^ cm/s).

On the other hand, nonmodified vancomycin, as well as the parent CPPs (**4**, **9**, **14**), were completely unable to penetrate the BBB (Table 3). In contrast, four conjugates—Van-PEG_3_-TP10 (**2**), Van-PEG_3_-PTD4 (**10**), Van-PEG_4_-PTD4 (**11**), and Van-Cystamine-PTD4 (**12**)—exhibited a slight tendency to diffuse through the artificial BBB. Among these, Van-PEG_3_-TP10 (**2**) showed the highest effective permeation coefficient (P*_e_* value of 0.187 × 10^−6^ cm/s).

The other compounds, including the four Van-Tat (47–57) (**5**–**8**) conjugates, were unable to cross the BBB. Although the Van-Tat (47–57) and Van-Arg_9_ conjugates (**15**–**17**) did not diffuse through the artificial BBB, they demonstrated relatively higher mass retention values (up to 78%) compared to the corresponding conjugates of Van with PTD4 or TP10. While these compounds were effectively absorbed by the membrane, they were ultimately unable to cross it.

## 3. Discussion

The use of Van in antibacterial therapy is hindered by several significant limitations. Its effectiveness is increasingly compromised due to the rise of multidrug-resistant clinical strains, such as MRSA, VISA, VRSA, and VRE [1,5,6,7,8]. In addition to the growing resistance to this antibiotic, vancomycin’s hydrophilic nature results in poor penetration into cells and specific regions of the body, such as the brain, limiting its effectiveness in treating central nervous system infections like meningitis [7,11,12]. Moreover, its use is associated with toxic effects. Consequently, modifying Van to enhance its antibacterial activity, reduce toxicity, and improve brain tissue penetration remains one of the key challenges in medicinal chemistry. Over the past few decades, various strategies have been developed to address these issues, including chemical modifications of Van’s structure, the synthesis of new derivatives, and conjugation with other chemical compounds [1,2,3,4,6,7,10,11,13,14,15,16,17,18,19,20,21]. One of the most promising strategies involves conjugating Van with cell-penetrating peptides (CPPs), which possess both penetrating and, at times, antimicrobial properties [7,15,17].

In this study, a series of vancomycin conjugates with CPPs, such as TP10, Tat (47–57), PTD4, and Arg_9_, were designed and synthesized. Given the presence of multiple reactive groups in both Van and CPP molecules, the CPPs were covalently attached to Van using the highly effective and chemoselective 1,3-dipolar Huisgen cycloaddition, commonly known as the “click reaction” [7]. Various linkers containing polyethylene glycol (PEG_3_ or PEG_4_) or a disulfide bridge (Cystamine or Suc-Cystamine) were used to modify the Van molecule. The PEG_4_ and Suc-Cystamine linkers were coupled to the primary amino group in Van’s carbohydrate moiety via an amide bond, while the PEG_3_ and Cystamine linkers were coupled via an amide bond to Van’s *C*-terminal carboxyl group. These modification positions are considered nonbinding sites, and therefore, generally do not affect Van’s binding affinity to the terminal residues of lipid II [4,6].

All linkers were equipped with azido groups at their ends, enabling a “click reaction” with propiolate groups attached to the *N*-terminal amino groups of CPPs. This reaction formed a chemically stable 1,2,3-triazole ring. The linkers were chosen for their low cytotoxicity, good solubility, neutral overall charge, and ability to conjugate Van to CPPs without disrupting the penetrating properties of the CPPs or the antibacterial activity of Van. A key advantage of linkers containing a disulfide bridge is the potential for Van molecules to be released inside cells upon reduction of the disulfide bond, further enhancing drug delivery.

The obtained conjugates and their CPP components were evaluated for antimicrobial activity against various bacterial strains, cytotoxicity, and their ability to permeate the BBB. As expected, all conjugates exhibited antibacterial activity against staphylococcal and enterococcal strains. *E. coli* and *P. aeruginosa* strains were completely resistant to treatment with the synthesized compounds, except for TP10 and its Van conjugates. Among the synthesized compounds, the Van-Tat (47–57) and Van-Arg_9_ conjugates demonstrated the highest inhibitory activity. Specifically, two conjugates, Van-PEG_3_-Tat (47–57) (**5**) and Van-Cystamine-Tat (47–57) (**7**) were significantly more active than Van against the reference strain of *S. aureus* (both with 17-fold lower MICs) and *E. faecalis* (with approximately 36-fold and 9-fold lower MICs, respectively). Moreover, they showed high activity against the clinical VRE strain, with MIC values approximately 52.5-fold and 6.5-fold lower than gentamicin, respectively. In contrast, Van-Suc-Cystamine-Tat (47–57) (**8**) exhibited about 1.7-fold higher activity against MRSA.

The Van-PEG_3_-Arg_9_ conjugate (**15**) was more active than Van against the MRSA strain (with over 6-fold lower MIC), the reference strain of *E. faecalis* (approximately 8.5-fold lower MIC), and the clinical VRE strain (with an MIC approximately 25-fold lower than gentamicin). Another conjugate, Van-PEG_4_-Arg_9_ (**16**), demonstrated higher antibacterial activity than Van against both reference strains of *S. aureus* and *E. faecalis*, as well as the clinical MRSA strain (with approximately 17-fold, 34-fold, and 13-fold lower MICs, respectively). However, the Van-PEG_3_-TP10 (**2**) conjugate showed only a slightly lower MIC compared to Van against the reference *S. aureus* strain, and against *E. coli*, it was the only conjugate with an MIC about 4-fold lower than gentamicin.

It is worth noting that the CPP components of the conjugates (except PTD4, **10**–**13**) also exhibited moderate antibacterial activity against the tested strains, usually with MIC values higher than those of Van or gentamicin. Therefore, the possible interactions between Van and CPPs were investigated. The calculated FICI values against the reference strain of *S. aureus* indicate that neither synergy nor antagonism occurs between Van and Prop-Tat (47–57) (**4**) or Van and Prop-Arg_9_ (**14**). In our previous studies, we demonstrated that there is no interaction between Van and TP10 against different *S. aureus* strains, so it was not tested in this study [7]. However, the possible interaction between Van and PTD4 was not investigated due to the complete inactivity of this CPP against the tested strains. These studies suggest that there is no interaction between Van and the CPP components of the conjugates.

To better understand the phenomenon of the diverse antimicrobial activity of Van-CPP conjugates, a cytotoxicity test (using the Neutral Red cytotoxicity assay) and a BBB permeability test (using the PAMPA–BBB assay) were conducted for selected compounds. The cytotoxicity of the Van-CPP conjugates and their components was evaluated against normal human fibroblast cells. This cell line was found to be sensitive to Van, with concentrations above 32 µg/mL significantly reducing cell viability to approximately 22% (a decrease similar to the effect of the 10% DMSO control). In all cases, the combination of Van with CPPs resulted in reduced cytotoxicity compared to Van alone, even though one of the CPP components, TP10 (**1**), exhibited higher cytotoxicity than Van at concentrations above 32 µg/mL. Overall, most conjugates and their CPP components, particularly Van conjugates with Tat (47–57) (**5**–**7**) and Arg_9_ (**15**–**17**), did not show a stronger impact on cell viability across the concentration range tested. Among the synthesized conjugates, the strongest cytotoxicity was demonstrated by the Van-PEG_3_-TP10 conjugate (**2**) at concentrations above 64 µg/mL, which was significantly above its MIC values. PTD4 (**9**) and its conjugates (except Van-Cystamine-PTD4, **12**) also affected cell viability, but their cytotoxicity was much lower than that of TP10 (**1**) and its conjugates (**2**, **3**).

In contrast to the cytotoxicity results, compounds with the highest antibacterial activity and lowest cytotoxicity, such as the Van-Tat (47–57) (**5**–**8**) and Van-Arg_9_ conjugates (**15**–**17**), were completely unable to cross the artificial BBB in the PAMPA–BBB assay. Similarly, nonmodified Van also did not diffuse through the artificial BBB. Interestingly, these conjugates exhibited relatively high mass retention values (up to approximately 78%), suggesting that they may be effectively absorbed by the membrane. On the other hand, Van-PEG_3_-TP10 (**2**), Van-PEG_3_-PTD4 (**10**), Van-PEG_4_-PTD4 (**11**), and Van-Cystamine-PTD4 (**12**) conjugates, which showed moderate antibacterial activity and higher cytotoxicity, demonstrated a very low ability to penetrate the BBB.

It is important to note that the PAMPA–BBB assay, based on an artificial BBB, considers only passive transport mechanisms, such as transcellular passive diffusion. It does not account for active transport processes, including active influx and efflux transporters, which may significantly affect the real ability of compounds to cross the BBB in in vivo conditions. Additionally, the partial or complete metabolism of compounds, which could limit their BBB permeability, should also be considered.

Based on our observations, it appears that a relationship exists between the biological activity of Van-CPP conjugates and the physicochemical properties of their CPP components. Van conjugates with Tat (47–57) (**5**–**8**) and Arg_9_ (**15**–**17**), which had the highest net charges (+8 and +9, respectively) and the lowest hydrophobicity, demonstrated the highest antibacterial activity, the lowest cytotoxicity, and the lowest ability to cross the artificial BBB. As shown in Table 3, the calculated net charge values, hydrophobicity indexes of the CPP components, and retention times of the conjugates from HPLC analyses support this hypothesis.

On the other hand, Van conjugates with PTD4 (**10**–**13**) and TP10 (**2**, **3**), which had lower net charges (+3 and +4, respectively) and higher hydrophobicity in their CPP components, exhibited moderate antibacterial activity, higher cytotoxicity, and greater BBB permeability compared to the aforementioned conjugates. This suggests that the net charge of the CPP component is more critical for the antibacterial activity of Van-CPP conjugates, whereas the hydrophobicity of the CPP component plays a more important role in cytotoxicity and BBB permeability.

However, it remains challenging to definitively establish a correlation between the conjugates’ activity and the type of linker used or its attachment site in the Van structure. Conjugates containing polyethylene glycol (PEG_3_ or PEG_4_) linkers seem to show slightly higher antibacterial activity than those containing a disulfide bridge (Cystamine or Suc-Cystamine). This difference may be linked to the lower chemical stability of disulfide bridges, which are more prone to degradation through reduction.

These findings are consistent with our previous studies involving various Van-TP10 conjugates [7]. Both the current and past results confirm that conjugating Van with CPPs can improve its pharmacological properties by enhancing antibacterial activity, reducing cytotoxicity, and increasing BBB permeability. Our recent research demonstrated that Van-TP10 conjugates exhibited increased antibacterial activity compared to unmodified Van against clinical strains of MRSA, h-VISA, and, in some cases, VRE. Notably, these conjugates showed antibacterial activity against intracellular MRSA in HEK293 cells and improved access to brain tissue in an in vivo mouse model, all without significantly increasing toxicity (as confirmed by an erythrocyte lysis assay).

Meanwhile, the current PAMPA–BBB assay results for Van conjugates with Tat (47–57) (**5**–**8**) and Arg_9_ (**15**–**17**) are somewhat disappointing. Despite their high antibacterial activity and negligible cytotoxicity across a wide range of tested concentrations, they were unable to cross the BBB. It is possible that better results for these conjugates could be achieved in an in vivo model for BBB penetration. The low BBB permeability of these compounds may be partly explained by the high positive net charge of their CPP components. In previous studies, molecular dynamics simulations of the Tat peptide interacting with membranes revealed that the high positive charge of Tat favors strong electrostatic interactions, leading to its adsorption on the membrane surface [29]. The high energy of these interactions, combined with unfavorable interactions between the hydrophilic peptide and the membrane’s hydrophobic interior, prevented Tat from crossing the membrane barrier.

In summary, our studies have demonstrated that conjugating Van with CPPs, particularly Tat (47–57), oligoarginine (Arg_9_), and TP10, can significantly enhance its antimicrobial activity against Gram-positive bacteria, including *S. aureus* and *Enterococcus* spp., while reducing its cytotoxicity and potentially improving its access to brain tissues. However, further research is necessary to investigate these conjugates’ effects on *S. aureus* biofilm formation, activity against intracellular strains, susceptibility to degradation, and tissue penetration, including their ability to cross the BBB, using more precise in vivo models. We conclude that these findings provide a strong basis for the design of novel antimicrobial agents effective in the treatment of infections caused by drug-resistant staphylococcal and enterococcal strains, as well as their ability to cross the BBB.

## 4. Materials and Methods

### 4.1. Reagents

All reagents and solvents were of analytical, HPLC, or LC-MS grade and obtained from Sigma-Aldrich Co. (Poznań, Poland). Solutions were freshly prepared using distilled deionized water from a Milli-Q Millipore system (Bedford, MA, USA) and filtered through a 0.22 μm filter before use. Fmoc (fluorenyl-9-methoxycarbonyl)-protected l-amino acids for peptide synthesis were sourced from Bachem AG (Bubendorf, Switzerland). Rink-Amide TentaGel S RAM resin was purchased from Rapp Polymere GmbH (Tuebingen, Germany). 2-((2-azidoethyl)disulfanyl)ethanamine hydrochloride (N_3_-Cystamine · HCl) and 4-(2-((2-azidoethyl)disulfanyl)ethylamino)-4-oxobutanoic acid succinimidyl ester (N_3_-Cystamine-Suc-NHS) were obtained from Iris Biotech GmbH (Marktredwitz, Germany). 15-azido-4,7,10,13-tetraoxapentadecanoic acid *N*-hydroxysuccinimidyl ester (N_3_-PEG_4_-NHS) and 1-amino-11-azido-3,6,9-trioxoundecane (N_3_-PEG_3_-NH_2_) were purchased from ChemPep Inc. (Wellington, FL, USA). The lipid mixture from porcine polar brain lipid extract was purchased from Avanti Polar Lipids (Alabaster, AL, USA). The donor filtration plate (Multiscreen Filter Plate with PVDF filter) and the acceptor plate (Multiscreen Receiver Plate) were obtained from Merck (Warsaw, Poland).

### 4.2. Synthesis of Peptides

All peptides and their alkyne-functionalized analogs were synthesized using solid-phase peptide synthesis (SPPS) on an automatic peptide synthesizer (Quartet, Protein Technologies, Tucson, AZ, USA) following the Fmoc strategy [7]. TentaGel S RAM resin (loading 0.25 mM/g) was used as the starting material. Fmoc-protected amino acids were activated with a 3-fold molar excess of *O*-(benzotriazole-1-yl)-1,1,3,3-tetramethyluronium tetrafluoroborate (TBTU), along with *N*-hydroxybenzotriazole (HOBt) and *N*-methylmorpholine (in a molar ratio of 1:1:2) in *N,N*-dimethylformamide (DMF) for 2 × 0.5 h. After the peptide backbone was synthesized, the *N*-terminal Fmoc group was removed with 20% piperidine in DMF (2 × 3.5 min), and the propiolate group (Prop) was attached to the *N*-terminal amino group using a 10-fold molar excess of propiolic anhydride in DMF for 1.5 h at room temperature. Propiolic anhydride was prepared by mixing *N,N′*-diisopropylcarbodiimide (DIC), and propiolic acid (in a molar ratio of 1:2) in DMF. This mixture was stored at 0 °C for 10 min before being added to the reaction vessel containing the peptidyl resin.

Once the reaction was complete, the resin was washed with dichloromethane and dried in a vacuum desiccator. Peptides were cleaved from the resin and deprotected using a mixture of trifluoroacetic acid (TFA), phenol, triisopropylsilane, and water (96/5/2/5, *v*/*v*/*v*/*v*) for 3 h at room temperature under an inert gas (argon). The peptides were then precipitated with cold diethyl ether, filtered, dissolved in water, and lyophilized.

Crude peptides were purified using a preparative HPLC system (SpotPrep II, Armen, Brittany, France) on a Reprosil 100 C-18 column (Dr. Maisch GmbH, 40 × 250 mm, 10 µm particle size), employing several gradients of ACN with 0.08% TFA at a flow rate of 25 mL/min. Fractions were analyzed using an analytical UHPLC-MS system (Shimadzu Nexera X2, Tokyo, Japan) with a ReproSil Pure 120 ODS-3 column (Dr. Maisch GmbH, 100 × 2 mm, 2.4 µm particle size) and several gradients of ACN with 0.1% formic acid (FA) and 0.05% TFA at a flow rate of 0.3 mL/min. The elution was monitored using a UV detector at 220 nm and a mass spectrometry detector (Shimadzu LCMS-2020). Fractions with an HPLC purity >95% were collected and lyophilized. The molecular mass of the synthesized peptides was confirmed by electrospray ionization mass spectrometry (ESI-MS).

Table 4 presents the physicochemical properties of the synthesized compounds, including their calculated molecular weights, observed ions ([*m*/*z*]), and yields.

### 4.3. Synthesis of Vancomycin Derivatives

The azido-functionalized Van derivatives (Van-PEG_3_-N_3_, Van-PEG_4_-N_3_, Van-Cystamine-N_3,_ and Van-Suc-Cystamine-N_3_) were synthesized in solution [7]. For Van-PEG_4_-N_3_ and Van-Suc-Cystamine-N_3_, 15-azido-4,7,10,13-tetraoxopentadecanoic acid succinimidyl ester (N_3_-PEG_4_-NHS) or 4-(2-((2-azidoethyl)disulfanyl)ethylamino)-4-oxobutanoic acid succinimidyl ester (N_3_-Cystamine-Suc-NHS) was attached via an amide bond to the primary amino group in the carbohydrate moiety of vancomycin hydrochloride, dissolved in water with the addition of *N,N*-diisopropylethylamine (DIPEA) (molar ratio 1.5:1:3). The mixtures were stirred at 4 °C for 0.5 h. Upon completion of the reaction, the desired products were immediately separated using preparative HPLC.

For Van-PEG_3_-N_3_ and Van-Cystamine-N_3_, derivatives were obtained by attaching 1-amino-11-azido-3,6,9-trioxoundecane (N_3_-PEG_3_-NH_2_) or 2-((2-azidoethyl)disulfanyl)ethanamine hydrochloride (N_3_-Cystamine · HCl) to the *C*-terminal carboxyl group of vancomycin hydrochloride via an amide bond. These reactions were performed in DMF using 2-(1*H*-7-azabenzotriazole-1-yl)-1,1,3,3-tetramethyluronium hexafluorophosphate (HATU) and DIPEA (molar ratio 1:1.2:1.2:4.1), with the mixtures stirred at room temperature for 1 h. Afterward, the reaction products were immediately separated using preparative RP-HPLC.

The crude products were purified using a preparative HPLC system (SpotPrep II, Armen) with a Reprosil 100 C-18 column (Dr. Maisch GmbH, 40 × 250 mm, 10 µm particle size). Several gradients of ACN with 0.08% TFA were applied at a flow rate of 25 mL/min for purification. Eluates were fractionated and analyzed by an analytical UHPLC-MS system (Shimadzu Nexera X2 with LCMS-2020 detector) using a ReproSil Pure 120 ODS-3 column (Dr. Maisch GmbH, 100 × 2 mm, 2.4 µm particle size) with several gradients of ACN containing 0.1% FA and 0.05% TFA at a flow rate of 0.3 mL/min. The elution was monitored with a UV detector set at 220 nm and a mass spectrometry detector. Fractions containing the desired products with HPLC purity >95% were collected and lyophilized. The identities of the products were confirmed using ESI-MS.

### 4.4. Conjugation of Vancomycin with CPPs

Conjugates of Van with CPPs (Figure 1) were synthesized using the “click reaction”—a Cu(I)-catalyzed specific 1,3-dipolar Huisgen cycloaddition reaction [7]. The reactions involved alkyne-functionalized CPP analogs (Prop-TP10, Prop-Tat (47–57), Prop-PTD4, and Prop-Arg_9_) with azido-functionalized Van derivatives (Van-PEG_3_-N_3_, Van-PEG_4_-N_3_, Van-Suc-Cystamine-N_3_, and Van-Cystamine-N_3_ in a water/*tert*-butanol medium (1:1 *v*/*v*) in the presence of 0.1 M CuSO_4_ × 5H_2_O and a freshly prepared 0.5 M sodium ascorbate solution (molar ratio 1.5:1:2:5). The reaction mixtures were stirred at room temperature for approximately 4 h. Once the 1,2,3-triazole formation reactions were complete, the solvents were evaporated, and the products were lyophilized.

The crude conjugates obtained were purified using a preparative HPLC system (SpotPrep II, Armen) with a Reprosil 100 C-18 column (Dr. Maisch GmbH, 20 × 250 mm, 10 µm particle size). Several gradients of ACN with 0.08% TFA at a flow rate of 12 mL/min were used for purification. The purity of the conjugates was verified using an analytical UHPLC-MS system (Shimadzu Nexera X2 with LCMS-2020 detector) with a ReproSil Pure 120 ODS-3 column (Dr. Maisch GmbH, 100 × 2 mm, 2.4 µm particle size) and several gradients of ACN with 0.1% FA and 0.05% TFA at a flow rate of 0.3 mL/min. The HPLC purity of the conjugates was established to be >95%, and the molecular mass of the synthesized compounds was confirmed by mass spectrometry (Table 4).

**Figure 3:**
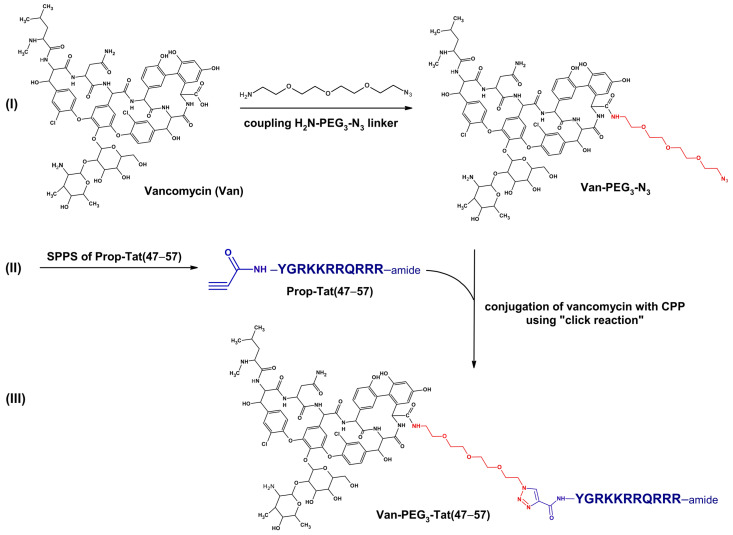
The general scheme of the synthesis of vancomycin conjugates with CPPs showing the preparation of one of the representatives of the conjugates—Van-PEG*_3_*-Tat (47–57). The syntheses consist of three main steps. (**I**) In the first step, azido-functionalized Van derivatives are obtained by coupling vancomycin with bifunctional linkers, e.g., H*_2_*N-PEG*_3_*-N*_3_* (1-amino-11-azido-3,6,9-trioxoundecane); (**II**) the second step is the solid-phase synthesis (SPPS) of CPP derivatives with a propiolate group attached to the *N*-terminus, e.g., Prop-Tat (47–57); (**III**) in the third step, vancomycin derivatives are conjugated with CPP derivatives using the highly effective and chemoselective 1,3-dipolar Huisgen cycloaddition, commonly known as the “click reaction”, which leads to the formation of a 1,2,3-triazole ring and stable Van-CPP conjugates, e.g., Van-PEG*_3_*-Tat (47–57).

Figure 3 shows the general scheme of the synthesis of vancomycin conjugates with cell-penetrating peptides.

### 4.5. Antimicrobial Activity Assay

The antimicrobial activity of the synthesized compounds was evaluated against four reference bacterial strains: *Staphylococcus aureus* (ATCC 29213), *Enterococcus faecalis* (ATCC 29212), *Escherichia coli* (ATCC 25922), and *Pseudomonas aeruginosa* (ATCC 27853), which are commonly used for determining antibacterial activity. In addition, two clinical strains, *S. aureus* (MRSA) and *E. faecium* (VRE), were included in the study. The bacterial strains were inoculated from Selectrol discs (Biomaxima SA, Lublin, Poland) onto blood agar (Graso Biotech, Starogard Gdański, Poland) and incubated at 37 °C for 24 h. Before testing, the strains were cultured aerobically on blood agar at 37 °C for 24 h.

The susceptibility of microorganisms to the synthesized compounds was determined using the broth microdilution assay, following the guidelines outlined by CLSI [30]. The final concentrations of the tested compounds in Mueller–Hinton broth, in 96-well plates (VWR International Sp. z o.o., Gdańsk, Poland), ranged from 0.125 to 256 mg/L. To prepare the bacterial suspension, bacteria from the overnight culture on blood agar were diluted in sterile 0.9% NaCl to achieve a turbidity equivalent to a 0.5 McFarland standard. The adjusted inoculum suspension was further diluted in Mueller–Hinton broth so that after inoculation, each well contained approximately 5 × 10^5^ CFU/mL. Aliquots (5 µL) of bacterial suspension were added to each solution. The sterility control (containing the broth) and the growth control (containing the reference strain in broth without the tested compound) were also set up. The antibiotics vancomycin and gentamicin were used as a control. The plates were incubated at 37 °C under aerobic conditions. The results are expressed as the MIC of the tested compounds, which was defined as the lowest concentration at which no visible growth of bacteria (no turbidity) was observed. The assay was performed in triplicate.

### 4.6. Determination of Interactions Between Van and CPP Components of the Conjugates

To determine possible interactions between Van and the CPP components of the conjugates, fractional inhibitory concentrations (FICs) were calculated for the tested compounds (Prop-Tat (47–57) and Prop-Arg_9_) using the following formula: FIC_A_ = MIC_A(with B)_/MIC_A_, FIC_B_ = MIC_B(with A)_/MIC_B_. To determine MIC_A(with B)_ and MIC_B(with A)_, a checkerboard assay was carried out in 96-well microtiter plates. Each test was performed in duplicate. The concentration range was 1.07 to 8 µg/mL for Van, 2.13 to 16 µg/mL for Prop-Tat (47–58), and 8.54 to 64 µg/mL for Prop-Arg_9_. Aliquots of 5 μL of bacterial suspension (at a concentration of 10^7^ CFU/mL) were added to each well of the 96-well plate containing 100 μL of test compounds in Mueller–Hinton broth. The bacterial strain used was *S. aureus* (ATCC 29213). The plates were then incubated at 37 °C for 24 h. After incubation, MIC_Van+Prop-Tat (47–57)_ and MIC_Prop-Tat (47–57)+Van_ were read at MIC of Prop-Tat (47–57) (8 μg/mL) and MIC of Van (2 μg/mL), respectively. Similarly, MIC_Van+Prop-Arg9_ and MIC_Prop-Arg9+Van_ were read at MIC of Prop-Arg_9_ (32 μg/mL) or MIC of Van (2 μg/mL), respectively. FIC values for Van, Prop-Tat (47–57) and Prop-Arg_9_ were calculated as follows: FIC_Van_ = MIC_Van+Prop-Tat (47–57)_ or FIC_Van_ = MIC_Van+Prop-Arg9_ divided by MIC of Van alone, FIC_Prop-Tat (47–57)_ = MIC_Prop-Tat (47–57)+Van_ divided by MIC of Prop-Tat (47–57) alone, and FIC_Prop-Arg9_ = MIC_Prop-Arg9+Van_ divided by MIC of Prop-Arg_9_ alone. The FIC indexes (FICIs) were calculated for the tested compounds as the sum of FIC_A_ and FIC_B_. The obtained FICIs were interpreted according to the following principle: FICI ≤ 0.5 synergy; FICI > 0.5 and ≤4 indifference (no interaction); FICI > 4, antagonism [31].

### 4.7. Human Fibroblast Cell Culture

Normal human fibroblasts, BJ line (CRL-2522™), Eagle’s Minimum Essential Medium (EMEM), and fetal bovine serum (FBS) were obtained from ATCC (Manassas, VA, USA). Cells were grown following ATCC guidelines in an EMEM medium supplemented with 10% FBS at 37 °C in a humidified atmosphere of 95% air and 5% CO_2_ in a HeraCell 150 incubator (Heraeus, Hanau, Germany).

### 4.8. Neutral Red Cytotoxicity Assay

The assay was performed as per previously published protocols with slight modifications [32,33]. In brief, BJ cells were plated in 96-well tissue culture plates (Nest Scientific Biotechnology, Wuxi, China) at a density of 6 × 10^3^ cells per well and allowed to attach for 24 h in EMEM supplemented with 10% FBS. After the incubation period, the medium was removed, and 10% dimethyl sulfoxide (DMSO)-treated cells (Sigma-Aldrich, St. Louis, MO, USA) were used as a positive control, while nontreated cells served as a negative control.

The cells were treated with peptides suspended in EMEM medium supplemented with 10% FBS and incubated for 24 h at 37 °C in a humidified atmosphere of 95% air and 5% CO_2_. After the incubation, the supernatants were discarded and replaced with 100 µL of non-supplemented EMEM containing 0.33% neutral red (Sigma-Aldrich) diluted 1:40. The cells were incubated for an additional 2 h at 37 °C. Following incubation, the neutral red medium was removed, and the cells were washed with 100 µL of phosphate-buffered saline (PBS) per well.

To extract the dye into the solution, the cells were treated with 150 µL of a solution containing 50% ethanol (Alchem, Torun, Poland), 49% distilled H_2_O, and 1% acetic acid (Alchem, Torun, Poland) and incubated under shaking at 37 °C for 10 min. Absorbance was measured at 540 nm using a Synergy H1 plate reader (BioTek Instruments, Winooski, VT, USA). The assay was performed in triplicate to ensure reproducibility.

### 4.9. PAMPA-BBB Assay

The following protocol was used to determine the effective permeability coefficient (P*_e_*) of compounds through an artificial membrane [34,35,36,37]. This assay utilized a 96-well plate assembly consisting of a filter plate pre-coated with polar brain lipids (donor plate) and a receiver plate (acceptor plate). The phospholipid mixture used to coat the membrane was extracted from porcine polar brain lipids, with the following composition: 12.6% phosphatidylcholine, 33.1% phosphatidylethanolamine, 18.5% phosphatidylserine, 4.1% phosphatidylinositol, 0.8% phosphatidic acid, and 30.9% of other compounds, including cerebrosides, sulfatides, and pigments.

In each well of the donor filtration plate (Multiscreen Filter Plate, PVDF with a pore size of 0.45 µm), 5 µL of the phospholipid mixture in *n*-dodecane (20 mg/mL) was carefully applied to form the artificial membrane. After coating, 200 µL of phosphate-buffered saline (PBS at pH 7.4) containing 5% dimethyl sulfoxide (DMSO) as a co-solvent, and the test compound (at a concentration of 20 µM) was added to each well of the donor plate (in triplicate). Diclofenac and diazepam were used as low- and high-permeability controls, respectively. The compound-filled donor plate was then carefully placed on the acceptor plate (Multiscreen Receiver Plate) that had been prefilled with 300 µL of acceptor solution (PBS buffer at pH 7.4). The plate lid was then replaced and the resulting assembled donor–acceptor plates were incubated at room temperature for 20 h. After this time, the donor plate was removed and solutions from each well of both acceptor and donor plates were collected for further analysis. The concentrations of the tested compounds were determined by LC-MS. The analysis was conducted by UHPLC-MS system (Shimadzu Nexera X2 with LCMS-2020 detector) using a ReproSil Pure 120 ODS-3 column (Dr. Maisch GmbH, 100 × 2 mm, 2.4 µm particle size) with several gradients of ACN with 0.1% FA and 0.05% TFA at a flow rate of 0.3 mL/min. The eluted solution was monitored by an ESI-MS detector operated in a selected ion monitoring mode (SIM). The results are expressed as the effective permeability coefficient (P*_e_*) for each compound calculated from the following formula [34]:

$$P_{e}=\frac{V_{D}\times V_{A}}{\left(V_{D}+V_{A}\right)\times A\times t}\times -\ln\left(1-\frac{C_{A}}{C_{E}}\right)$$

where P*_e_* is the effective permeability coefficient (cm/s), *V*_D_ is the volume of the donor compartment (0.2 cm^3^), *V*_A_ is the volume of the acceptor compartment (0.3 cm^3^), *A* is the effective filter area (0.3 cm^2^), *t* is the incubation time for the assay (72,000 s), *C*_A_ is the concentration of the compound in the acceptor compartment after the assay, *C*_E_ is the concentration of compound at theoretical equilibrium (i.e., the resulting concentration if the donor and acceptor solutions were simply combined, calculated from the formula below), *C*_D_ is the concentration of the compound in the donor compartment after the assay.

$$C_{E}=\frac{C_{D}\times V_{D}+C_{A}\times V_{A}}{V_{D}+V_{A}}$$

To determine the amount of the compound lost during the permeability measurement (as a result of binding to the plastic surface and/or retaining in the artificial membrane) mass retention (*R*) was calculated using the following formula [35]:

$$R=1-\frac{C_{D}\times V_{D}+C_{A}\times V_{A}}{C_{0}\times V_{D}}$$

where *C*_0_ is the initial concentration of the compound in the donor compartment.

## Figures and Tables

**Table 1:** Antimicrobial activity of the examined compounds against reference and clinical (MRSA and VRE) bacterial strains.

No.	Compound	MIC									
		*S. aureus ^a^* ATCC 29213		*S. aureus ^b^* MRSA		*E. faecalis ^a^* ATCC 29212		*E. faecium ^c^* VRE		*E. coli ^a^* ATCC 25922	
		µg/mL	µM	µg/mL	µM	µg/mL	µM	µg/mL	µM	µg/mL	µM
	Gentamicin	0.24	0.50	0.48	1.00	7.80	16.3	7.80	16.3	1.95	4.08
	Vancomycin	2	1.35	1.5	1.01	8	5.38	>256	>172	nd	nd
**1**	TP10	32	14.7	8	3.67	64	29.3	nd	nd	32	14.7
**2**	Van-PEG_3_-TP10	4	1.03	>256	>66	16	4.12	nd	nd	4	1.03
**3**	Van-PEG_4_-TP10	16	4.04	16	4.04	16	4.04	nd	nd	32	8.09
**4**	Prop-Tat (47–57)	8	4.97	32	19.9	256	159	32	19.9	128	79.5
**5**	Van-PEG_3_-Tat (47–57)	0.25	0.077	16	4.91	0.5	0.15	1	0.31	>256	>78.5
**6**	Van-PEG_4_-Tat (47–57)	1	0.30	4	1.20	4	1.20	128	38.5	>256	>77
**7**	Van-Cystamine-Tat (47–57)	0.25	0.078	8	2.48	2	0.62	8	2.48	>256	>79.5
**8**	Van-Suc-Cystamine-Tat (47–57)	1	0.30	2	0.60	4	1.20	128	38.5	>256	>77
**9**	Prop-PTD4	>256	>204	>256	>204	>256	>204	>256	>204	>256	>204
**10**	Van-PEG_3_-PTD4	4	1.38	8	2.76	16	5.51	256	88	>256	>88
**11**	Van-PEG_4_-PTD4	4	1.34	8	2.69	16	5.37	>256	>86	>256	>86
**12**	Van-Cystamine-PTD4	8	2.79	2	0.70	8	2.79	64	22.4	128	44.5
**13**	Van-Suc-Cystamine-PTD4	8	2.70	nd	nd	32	10.8	nd	nd	nd	nd
**14**	Prop-Arg_9_	32	21.7	4	2.71	128	87	32	21.7	64	43
**15**	Van-PEG_3_-Arg_9_	>256	>82	0.5	0.16	2	0.64	2	0.64	>256	>82
**16**	Van-PEG_4_-Arg_9_	0.25	0.078	0.25	0.078	0.5	0.156	64	20	>256	>80
**17**	Van-Cystamine-Arg_9_	4	1.30	nd	nd	8	2.59	nd	nd	>256	>83

*^a^* reference strain; *^b^* methicillin-resistant strain (clinical isolate no 203); *^c^* vancomycin-resistant strain (clinical isolate UCK 7979384), nd—not determined; Prop—propiolate group.

**Table 2:** Antimicrobial activity of Van and Prop-Tat (47–57) or Van and Prop-Arg_9_ in combination.

Combination of Compounds	MIC [µg/mL]				FICI *^a^*	Interpretation
	MIC_A_	MIC_A(with B)_	MIC_B_	MIC_B(with A)_		
Van and Prop-Tat (47–57)	2	4.50	8	12	3.75	indifference
Van and Prop-Arg_9_	2	3.30	32	27	2.53	indifference

MIC_A_—Van; MIC_B_—Prop-Tat (47–57) or Prop-Arg_9_; MIC_A(with B)—_Van with Prop-Tat (47–57) or Prop-Arg_9_; MIC_B(with A)—_Prop-Tat (47–57) or Prop-Arg_9_ with Van; *^a^* the FICI data were interpreted using the following criteria: FICI ≤ 0.5 synergy; FICI > 0.5 and ≤ 4 indifference; FICI > 4 antagonism.

**Table 3:** PAMPA–BBB permeability of selected compounds after 20 h of incubation.

No.	Compound	P*_e_* [10^−6^ cm/s]	R [%]	R_t_ [min]	H	Q
	Diazepam	14.8 ± 1.9	61.7 ± 6.4	6.75 *^b^*	N/A	N/A
	Diclofenac	3.44 ± 0.1	12.0 ± 3.3	8.53 *^b^*	N/A	N/A
	Vancomycin	0	11.3 ± 3.4	5.51 *^a^*	N/A	N/A
**2**	Van-PEG_3_-TP10	0.19 ± 0.03	10.0 ± 0.6	6.94 *^b^*	0.560	+4
**4**	Prop-Tat (47–57)	0	3.3 ± 0.7	3.48 *^a^*	−0.664	+8
**5**	Van-PEG_3_-Tat (47–57)	0	8.7 ± 3.8	5.95 *^a^*	−0.664	+8
**6**	Van-PEG_4_-Tat (47–57)	0	77.7 ± 0.3	6.41 *^a^*	−0.664	+8
**7**	Van-Cystamine-Tat (47–57)	0	23.8 ± 4.9	6.10 *^a^*	−0.664	+8
**8**	Van-Suc-Cystamine-Tat (47–57)	0	31.5 ± 1.5	6.31 *^a^*	−0.664	+8
**9**	Prop-PTD4	0	6.7 ± 1.5	5.97 *^a^*	0.053	+3
**10**	Van-PEG_3_-PTD4	0.010 ± 0.003	2.0 ± 0.6	7.12 *^a^*	0.053	+3
**11**	Van-PEG_4_-PTD4	0.066 ± 0.007	32.7 ± 0.9	7.66 *^a^*	0.053	+3
**12**	Van-Cystamine-PTD4	0.036 ± 0.004	12.7 ± 2.2	7.45 *^a^*	0.053	+3
**14**	Prop-Arg_9_	0	8.0 ± 1.2	2.98 *^a^*	−1.010	+9
**15**	Van-PEG_3_-Arg_9_	0	2.7 ± 0.9	5.44 *^a^*	−1.010	+9
**16**	Van-PEG_4_-Arg_9_	0	37.3 ± 4.8	5.89 *^a^*	−1.010	+9
**17**	Van-Cystamine-Arg_9_	0	20.7 ± 0.3	5.62 *^a^*	−1.010	+9

P*_e_*—the effective permeability coefficient: P*_e_* > 4.0 × 10^−6^ cm/s—indicative of high BBB permeation; P*_e_* from 4.0 × 10^−6^ cm/s to 2.0 × 10^−6^ cm/s—uncertain BBB permeation; P*_e_* < 2.0 × 10^−6^ cm/s—indicative of low BBB permeation; R—mass retention; P*_e_* and R values are presented as mean ± SEM from the experiment performed in triplicate; R_t_—UHPLC retention time: *^a^* grad. 10–50% ACN in 10 min, *^b^* grad. 30–100% ACN in 10 min; H—mean hydrophobicity of CPP component (according to the Fauchere and Pliska hydrophobicity scale); Q—net charge of CPP component at pH 7.4.

**Table 4:** Physicochemical properties of the synthesized compounds.

Compound	Calculated Molecular Weight	Observed Ions [*m*/*z*] (from ESI-MS Spectra)					Yield *^a^* [%]
		[M + H]^+^	[M + 2H]^2+^	[M + 3H]^3+^	[M + 4H]^4+^	[M + 5H]^5+^	
TP10	2181.81		1091.85	728.30	546.60		34
Prop-TP10	2233.79		1117.18	745.12	559.10		30
Prop-Tat (47–57)	1610.92		806.25	537.90	403.75		24
Prop-PTD4	1255.42	1256.30	628.55	419.30			47
Prop-Arg_9_	1474.77		738.20	492.45	369.60		24
Van-PEG_3_-N_3_	1649.53	1650.20	825.55				20
Van-PEG_4_-N_3_	1722.72	1723.45	862.10				7
Van-Cystamine-N_3_	1609.56	1610.40	805.55				46
Van-Suc-Cystamine-N_3_	1708.70	1710.00	855.50				14
Van-PEG_3_-TP10	3883.27			1294.30	971.65	777.60	48
Van-PEG_4_-TP10	3956.32			1318.40	989.05	791.45	85
Van-PEG_3_-Tat (47–57)	3259.65			1088.00	816.10	653.15	9
Van-PEG_4_-Tat (47–57)	3333.27			1111.65	834.20	667.55	23
Van-Cystamine-Tat (47–57)	3219.62			1074.10	805.90	644.90	11
Van-Suc-Cystamine-Tat (47–57)	3319.92			1107.50	830.85	664.80	4
Van-PEG_3_-PTD4	2904.00			969.10	727.20	581.80	43
Van-PEG_4_-PTD4	2976.90		1489.25	993.40	745.50	596.80	9
Van-Cystamine-PTD4	2863.80			955.80	717.25		77
Van-Suc-Cystamine-PTD4	2964.00		1483.05	989.00	742.05	594.55	3
Van-PEG_3_-Arg_9_	3123.87			1042.30	781.90	625.75	18
Van-PEG_4_-Arg_9_	3197.12			1066.85	800.20	640.40	16
Van-Cystamine-Arg_9_	3083.47			1028.80	771.95	617.75	7

*^a^* only fractions with HPLC purity above 95% were taken into account.

## Data Availability

All relevant data are included in the manuscript.
